# Microstructure and Mechanical Properties of the Joints from Coarse- and Ultrafine-Grained Al-Mg-Si Alloy Obtained via Friction Stir Welding

**DOI:** 10.3390/ma16186287

**Published:** 2023-09-19

**Authors:** Marta Lipińska

**Affiliations:** Faculty of Mechanical Engineering, Military University of Technology, Gen. S. Kaliskiego 2, 00-908 Warsaw, Poland; marta.orlowska@wat.edu.pl

**Keywords:** Al-Mg-Si alloy, severe plastic deformation, friction stir welding, microstructure, precipitation strengthening, mechanical properties

## Abstract

In the present study, the welding of coarse- (CG) and ultrafine-grained (UFG) Al-Mg-Si alloy using friction stir welding (FSW) was attempted. The purpose of welding the UFG material was to check the possibility of applying FSW to materials with a thermally unstable microstructure, which is achieved by severe plastic deformation. This group of materials has significant potential due to the enhanced mechanical properties as a result of the elevated number of structural defects. The CG sample was also examined in order to assess whether there is an influence of the base material microstructure on the weld microstructure and properties. To refine the microstructure, incremental equal channel angular pressing was used. Plastic deformation resulted in grain refinement from 23 µm to 1.5 µm. It caused an increase in the microhardness from 105 HV0.1 to 125 HV0.1 and the tensile strength from 320 MPa to 394 MPa. Similar welds obtained using an FSW method exhibited good quality and grain size in a stir zone of 5 µm. For both welds, a decrease in the microhardness occurred in the stir zone. However, for the weld of UFG Al-Mg-Si, the microhardness distribution was homogeneous, while for the weld of the CG, it was inhomogeneous, which was caused by different characteristics of the second-phase precipitates. The tensile strength of the welds was lowered and equaled 269 MPa and 220 MPa for the CG and UFG welds, respectively.

## 1. Introduction

Friction stir welding (FSW) [1] is a modern solid-state welding technique. In this process, a stable joint is obtained due to the mixing of plasticized materials, which occurs by the rotational and linear movement of a unique tool with a pin. As a result, the temperature increases to a point lower than the melting point of the welded materials. Thus, a dynamic recovery/recrystallization occurs, which changes the grain size, precipitates, or texture of the materials [2]. Due to its properties, such as its excellent mechanical properties of the welds, high repeatability, and efficiency, the FSW process has great potential in terms of its application, e.g., in the aerospace industry [3]. FSW has been intensively examined for various groups of materials, including aluminum (Al) and its alloys [4,5], steel [6], and magnesium [7]. Moreover, the FSW technique can also be used for joining thermally unstable materials such as ultrafine-grained (UFG) metals [8], as it is a solid-state welding technique where melting does not occur, and the heat input is lower than in fusion welding methods.

The application of FSW to UFG materials was examined mainly for Al [8]. In the case of commercially pure Al, grain growth from 1 µm for the base material (BM) to a few microns in the weld has been achieved [9,10]. It is caused by a dynamic recrystallization that occurs in the stir zone (SZ) [11]. This indicates grain coarsening compared to the UFG size regime [12]. As a result, the mechanical strength of the welds decreases compared to the UFG material, but a moderate increase when compared to annealed Al is observed. In the case of age-hardenable alloys, the second strengthening factor, i.e., precipitates, influences the mechanical properties in the SZ of the weld. When age-hardenable alloys with a coarse-grained (CG) microstructure are welded using the FSW method, a characteristic ‘W’ type of microhardness line across the weld is obtained. It is caused by various phenomena, which depend on the distance from the SZ, as different heat inputs and plastic deformation occur [13]. It was shown for Al alloys [14,15] that, when samples are welded in a peak hardened state, approaching the weld center, the precipitates are coarsened, dissolved, and dissolved with reprecipitation. As a result, changes in the microhardness are observed. The lowest hardness values are usually observed between the heat-affected zone (HAZ) and the thermomechanical-affected zone (TMAZ), where the dissolution of the precipitates is observed. The reprecipitation process causes a slight increase in the microhardness in the SZ. However, on the other hand, as shown for Al-Li alloy [16], FSW can result in an increase in the microhardness in the SZ, which is caused by the dissolution of equilibrium precipitates and the development of small strengthening precipitates. Based on this observation, it can be assumed that welding of the UFG material, which is age-hardenable, can be more beneficial in achieving better mechanical properties in the SZ than for commercially pure metals.

Therefore, the present study aims to investigate the potential of the FSW technique to weld precipitate-hardened Al alloy, which was plastically deformed. The welds were characterized regarding the microstructure evolution in the subsequent weld zones, and the correlation with the mechanical properties was analyzed. This allowed us to evaluate the changes in zones and estimate the potential of FSW as a technique that preserves the high mechanical properties of the thermally unstable base material.

## 2. Materials and Methods

### 2.1. Sample Preparation

The chemical composition of the investigated Al-Mg-Si alloy (EN AW-6082) [17] is presented in Table 1. Samples were delivered in peak hardened condition, i.e., T6. In this state, samples were subjected to a plastic deformation process. Incremental equal channel angular pressing (I-ECAP) [18,19] was applied as a severe plastic deformation (SPD) method. The angle of the die was equal to 90°; therefore, the strain imposed during one pass of I-ECAP equaled ε = 1.15. I-ECAP was performed at room temperature. In this process, the sample is deformed in a feed-shear sequence. The feeding stroke is under control, and in this study, the value of the feeding stroke was equal to 20% of the billet thickness, which gave 0.6 mm. This value was sufficient for overlapping the plastic deformation zones in subsequent cycles. The processed samples had the shape of a rectangular plate with dimensions of 100 × 62 × 3 mm. Plastic deformation was performed at Warsaw University of Technology, the Faculty of Mechanical and Industrial Engineering. Two similar welds were conducted on the FSW machine ESAB Legio 4UT with a tool with a conical pin with a diameter of 5–6 mm, which was 3 mm long. The rotational speed was 800 rpm, while the linear speed was 400 mm/min. The tool inclination angle equaled 2°. The weld from the CG samples (as-received condition) was denoted ‘CG weld’, and the weld from the UFG samples (after I-ECAP processing) was denoted ‘UFG weld’.

### 2.2. Microstructure Characterization

The light microscope Zeiss Axiovission (Oberkochen, Germany) was used for a general view of the welds. Observations were conducted on metallographic samples. For in-depth microstructure characterization, electron microscopy techniques were applied. The scanning electron microscope (SEM) Hitachi Su70 (Tokyo, Japan) equipped with an electron backscatter detector (EBSD) was used for the analysis of the microstructure features, such as grain size (d), the fraction of high- and low-angle grain boundaries (HAGB and LAGB, respectively), distribution of grain boundaries, microtexture components based on pole figures (PF), and grain necessary dislocation (GND) maps. GND was designated using the kernel average misorientation technique with a square kernel [20,21]. Observations on a transmission electron microscope (TEM), Jeol Jem 1200 (Tokyo, Japan), operating at 120 kV, were obtained to characterize the precipitates. Samples for SEM and TEM were prepared by electropolishing on a Struers Tenupol5 system using an A2 Struers electrolyte (Hovedstaden, Denmark). Electropolishing was performed at a voltage of 35 V and a temperature of 5 °C. Microstructure characterization was performed at the Faculty of Materials Science and Engineering, Warsaw University of Technology.

### 2.3. Mechanical Properties

Mechanical properties were investigated via microhardness measurements and static tensile tests. Microhardness tests were conducted using Vickers’s method with a 100 g (HV0.1) load on an EMCO DuraScan 80 G5 hardness device (London, ON, Canada). Maps of the microhardness were obtained with a step size of 350 µm in two directions, which resulted in a map with a number of measured points exceeding 1800. Maps were taken on the welds’ cross section. Static tensile tests were performed at room temperature with a constant displacement rate of 4 mm × min^−1^ and using a servo-hydraulic testing machine (INSTRON type 8802, Norwood, MA, USA). Digital image correlation (DIC) was used for strain calculation. The latter also allowed us to visualize the strain location during tensile tests in four states—initial, yield strength (YS), ultimate tensile strength (UTS), and before the rupture.

## 3. Results and Discussion

### 3.1. Microstructure Evolution

**Figure 1:**
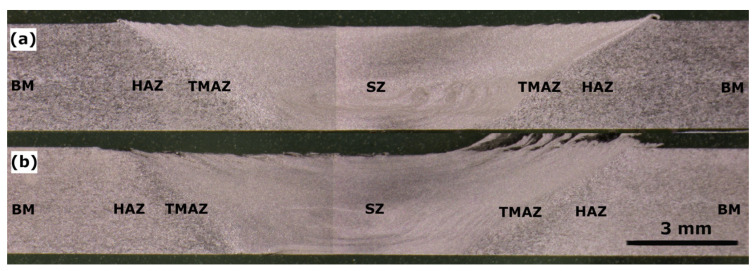
Macrographs of the (**a**) CG and (**b**) UFG welds (on the left—retreating side; on the right—advancing side).

The macrographs taken from the metallographic samples are shown in Figure 1, where all the regions, i.e., SZ, TMAZ, HAZ, and BM, are marked. Both welds were of good quality, with no defects observed. Also, characteristic features for the FSW process could be marked in both macrographs, such as the SZ in the center and the UFG weld on the advancing side, which is denoted by a flash. There were also differences in the grain size between zones. The BM and HAZ reveal bigger grains than the SZ for the CG weld. For the UFG weld, the differences in grain size are less pronounced, and further microstructural observations will show it more in detail.

**Figure 2:**
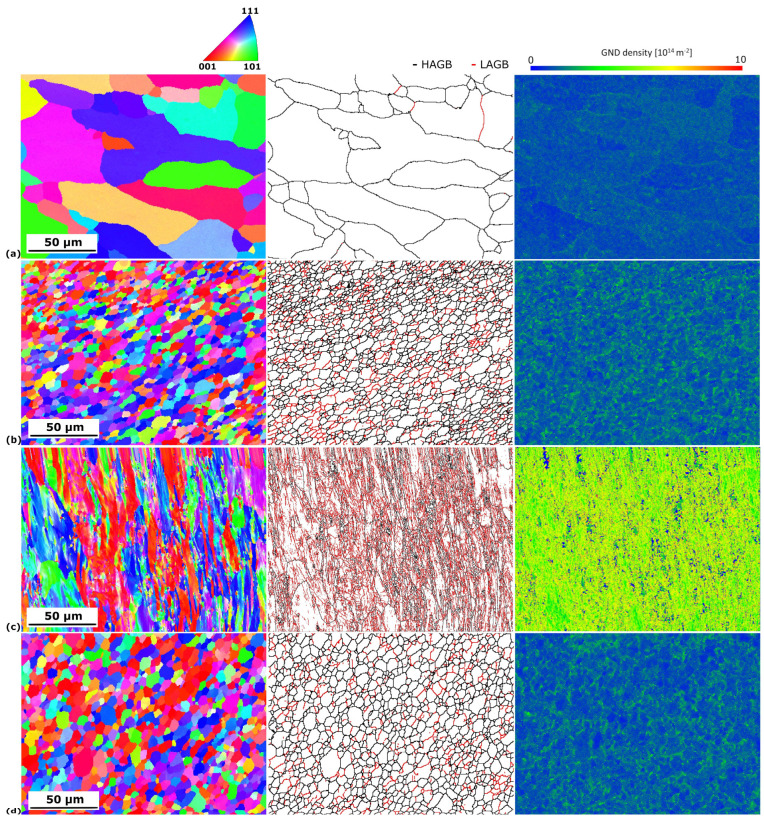
EBSD OIM of the (**a**) CG BM, (**b**) CG SZ, (**c**) UFG BM, and (**d**) UFG SZ.

**Figure 3:**
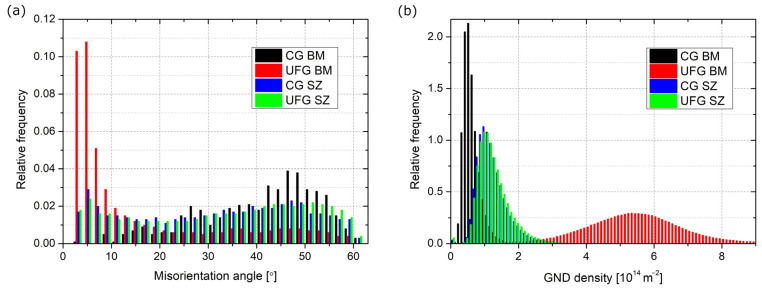
Graphs of (**a**) misorientation angle distribution and (**b**) GND density.

The results of the EBSD analysis are shown in Figure 2, where OIM, maps of the grain boundaries, and GND maps are presented. The misorientation angle distribution and GND density graphs are shown in Figure 3, while the quantitative data are gathered in Table 2. The average grain size of the CG BM equaled 23 µm, while for the UFG BM, this value was significantly reduced to 1.5 µm. The fraction of HAGB equaled 95 and 30%, respectively. Therefore, it can be stated that, even with one I-ECAP pass, distinct grain refinement occurred. The refinement occurred due to the formation of a subgrain structure, which is typical for low strain values in the case of Al and its alloys [22]. It can be seen in the majority of grains with LAGB (almost 70%), which are marked in red in Figure 2. It can also be observed in the graph in Figure 3a, where, for the UFG BM, the majority of grain boundaries have a misorientation angle below 10°. Higher strain values (a higher number of I-ECAP passes) would be required for further subgrain rotation and, thus, a higher fraction of HAGB [23]. In the case of the SZ, similar values of both grain size and the fraction of HAGB were obtained for both welds, and they equaled around 5 µm and 75–77%, respectively. This can be seen in the misorientation angle distributions (Figure 3a), which are almost identical for both SZs. The microstructure in the SZ indicates that continuous dynamic recrystallization (DRX) was the mechanism responsible for grain formation [24]. This is shown by equiaxial grains with a size of a few microns and the majority of grain boundaries being HAGB. However, a slightly higher grain size was observed for the SZ of the UFG weld. Also, a higher GND was maintained for the CG SZ. Nevertheless, the differences were not significant.

GND density is connected with grain size. It was shown that, during shearing, the initiation sites were at the triple joints or the grain boundaries, and then they propagated to the grain interiors [25]. GND was highly concentrated at the grain boundaries and was less so at the grain interiors. As presented for the Al-Mg-Si alloy, an increase in grain size during annealing resulted in a decrease in GND density [26]. Moreover, the highest density was observed for small grains, as they had a larger surface-area-to-volume ratio and played a more critical role in the nucleation of GND [27]. Therefore, with increasing shearing, the GND density increased. GND is a parameter that can be correlated with grain refinement and recrystallization. It explains the differences observed in the present study and those seen in Figure 2. The highest density of GND was observed for the UFG BM (Figure 3b), as this sample underwent plastic deformation. This value was the lowest for the CG BM, which is connected with the highest average grain size and a lack of subgrain structure. For the SZ, severe shearing occurred. However, due to the increase in temperature, DRX occurred. As a result, the GND density was reduced compared to the UFG BM, but was higher than for the CG BM.

**Figure 4:**
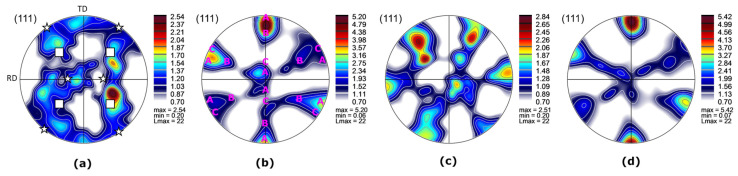
PF of the (**a**) CG BM (square—cube texture; star—Goss texture), (**b**) CG SZ (with marked identified shear components), (**c**) UFG BM, and (**d**) UFG SZ of the welds.

Texture was analyzed based on {111} PF, which is presented in Figure 4. For both welds, two regions were investigated—BM and SZ. For the CG BM, the texture intensity was not significant. However, rolling texture components were present, and they could be considered as a cube $\left\{001\right\}<100>$ and Goss {011} <100> [28]. They are marked in Figure 4a. For the remaining samples, similar shear texture components were identified. For simplification, they are only marked in Figure 4b. For the UFG BM, the shear texture components were inclined. Shear texture could be detected with components consisting of A $\left\{111\right\}<\mathrm{uvw}>$ and C $\left\{001\right\}<1\bar{1}0>$, with traces of B fiber components [29]. This is commonly observed in materials after FSW, as during the process, severe shearing occurs [30,31]. The texture was similar to UFG BM. The observed difference was a maximum intensity, which was higher for the SZ than for the UFG BM.

**Figure 5:**
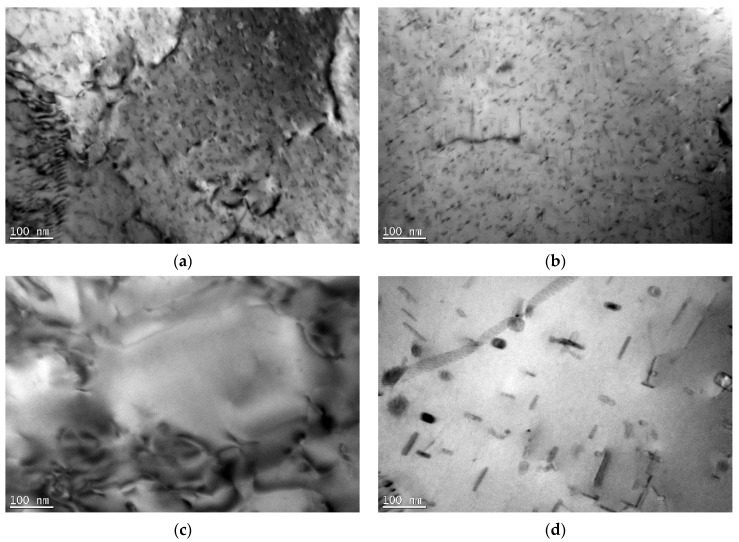
The TEM micrographs of the (**a**) BM (β″ precipitates), (**b**) HAZ (β″ precipitates), (**c**) SZ (lack of precipitates), and (**d**) SZ (β’ and β precipitates) of the weld from CG Al-Mg-Si alloy.

**Figure 6:**
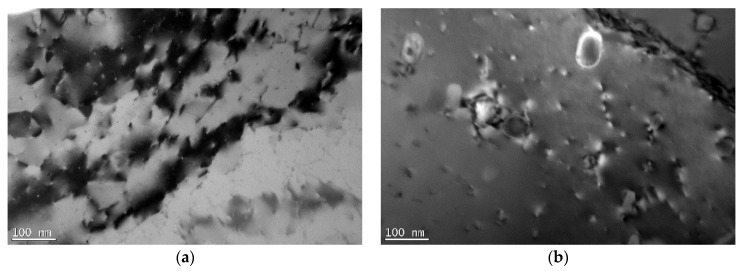

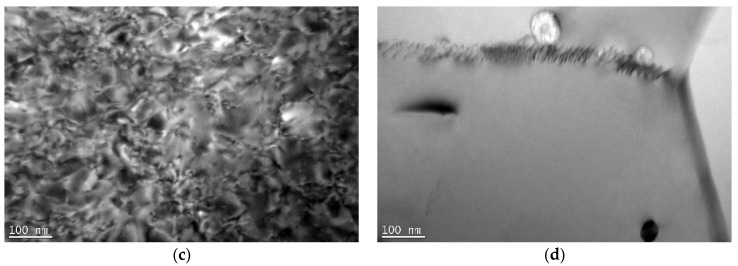
The TEM micrographs of the (**a**) BM (β″ precipitates), (**b**) HAZ (β″ precipitates—dissolving), and (**c**,**d**) SZ of the weld from UFG Al-Mg-Si alloy.

TEM micrographs of the BM and SZ are presented in Figure 5 for the CG weld and Figure 6 for the UFG weld. The CG BM (Figure 5a) revealed coarse grains with nano-sized precipitates. As the material was investigated in the T6 state, these precipitates were presumably β″ [32]. They were in the form of needles, with a length of approximately 50 nm and an orientation along the <100> direction [33]. In the SZ of the CG weld, various phenomena were observed with precipitates. Depending on the region in the SZ, they underwent dissolution (Figure 5c); however, in some, coarser precipitates were observed (Figure 5d). Based on the literature analysis, they can be assumed to be β’ (rods) and β (plates) [34,35], which contributed to the strengthening being lower in comparison to the β″ precipitates.

Between the welds, two main differences were observed. The first one was that, for the UFG BM, β″ precipitates were also observed; however, their size was reduced (Figure 6a) compared to the CG BM (Figure 5a). SPD processing may result in various phenomena that occur with second-phase precipitates, such as acceleration of precipitation [36], fragmentation due to severe shear forces [37], or dissolution [38]. Based on a comparison between Figure 5a and Figure 6a, two final processes could have taken place in the case of the I-ECAP process on the Al-Mg-Si alloy. Shearing of β″ precipitates is commonly observed. As shown in [39], with an increasing shear value, the final size of precipitates decreases as moving dislocations more frequently shear them. As a result, their shape changes from needle-like to more equiaxial, their length decreases, and their orientation may change. In the case of the UFG SZ, the precipitates dissolved in the whole zone (see Figure 6c,d). This is contrary to what was observed in the CG weld, where in the SZ, a variety of precipitates were observed. The reason for this may be the initial state of the precipitates, i.e., in the BM. For the UFG weld, the precipitates were smaller in the BM. During FSW, both plastic deformation and heat input influence the changes in the microstructure. In the case of the UFG weld, the size of the precipitates was smaller for the BM than for the CG weld. Therefore, the necessary driving force for dissolution was smaller. As a result, precipitates underwent dissolution.

In the SZ, many changes occurred, which are the result of dynamic recrystallization, which is commonly observed for Al and its alloys, as they have high stacking fault energy [11]. During FSW, due to the movement of the pin, intense plastic deformation occurs. As a result of the friction between the shoulder and the material, the temperature increases, but it does not exceed the melting point of the joined materials. These processes result in the occurrence of DRX, which completely reorganizes the microstructure. The changes are related to the grain size, fraction of HAGBs, texture components, and second-phase precipitates. Dynamic recrystallization results in the formation of new grains. Based on the changes in the microstructure in the SZ, three types of DRX could be distinguished—continuous (CDRX), discontinuous, and geometric recrystallization [40]. In the present study, for both welds, due to the equiaxial shape of the grain and the majority of HAGB, CDRX can be assumed to be a major DRX mechanism. CDRX occurs at high temperatures, and the formation of new grains occurs through the gradual increase in the misorientation angles of the subgrains. It was explained in more detail in the work [40]. It was observed that, during the formation of new grains in CDRX, in order to accommodate the plastic strain, the multiplicated dislocations tend to form dislocations tangles and walls, which subsequently transform into subgrains. Further deformation results in further rotation of the misorientation angle of such subgrains, and finally, new grains with HAGB are formed.

In the present study, the base materials differed significantly in the case of the grain size and fraction of HAGB and LAGB. However, in the SZ, a similar grain size and fraction of HAGBs were obtained. This indicates that the microstructure of the BM does not have an influence on the grain size, shape, and fraction of HAGB in the SZ. Also, the same texture components were observed for both SZs. In [41], it was shown that there is no dependency between the texture of the BM and the SZ. Similar observations were obtained in the present study, where the texture of the BM differed significantly; however, the same texture components were observed for both SZs. Therefore, it can be stated that, in this aspect, FSW parameters play a crucial role, and they influence the final microstructure.

In the case of second-phase precipitates, their state also changed in the SZ. However, in this aspect, heat input seemed to play a crucial role. The sequence of the precipitation in the Al-Mg-Si alloy is as follows: supersaturated solid solution -> GP zone -> β″ -> β′ -> β. Initially, for both BMs, β″ precipitates were present in the microstructure. For the UFG weld, precipitates dissolved completely, and in the SZ, it can be concluded that a supersaturated solid solution was obtained. For the CG weld, precipitates partially dissolved, but also coarsened precipitates were observed. The reason for this lies in the initial state of the precipitates: as for the UFG BM, their size was smaller, and thereby, presumably, the required driving force for their dissolution was smaller.

### 3.2. Mechanical Properties

**Figure 7:**
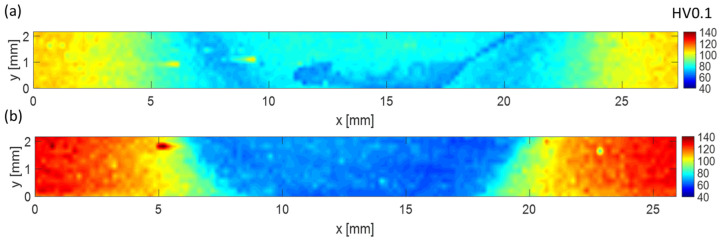
Microhardness maps of the cross section of the welds from (**a**) CG and (**b**) UFG Al-Mg-Si.

The results of the microhardness measurements are shown in Figure 7. The average microhardness value for the CG BM equaled 105 HV0.1, while for the UFG BM, it was 125 HV0.1. This indicates that one pass of I-ECAP was effective in hardening the material. In the HAZ, TMAZ, and SZ, a decrease in microhardness compared to the BM was obtained for both welds. The difference in the hardness value in the SZ was observed between the samples. The whole SZ was homogeneous for the weld from UFG Al-Mg-Si, and an average value of about 65 HV0.1 was measured, indicating an abrupt decrease compared to the BM. In the case of the weld from CG Al-Mg-Si, a drop was also observed, but higher values were obtained, as the average microhardness equaled 76 HV0.1. However, the microhardness distribution was heterogeneous, and more significant variations were observed.

The lowest microhardness values for precipitate-strengthening alloys were commonly observed in the HAZ [42]. This was caused by the changes in precipitates across the weld due to different thermomechanical characteristics. Regarding Al-Mg-Si alloys, for the AA6061 alloy, it was observed that FSW resulted in refined recrystallized grains with uniformly distributed dispersoids and the dissolution of primary strengthening precipitates β″ in the SZ, which resulted in a decrease in the microhardness. In addition, the lowest values were obtained in HAZ adjacent to TMAZ [20], where a substructure was observed, and the primary precipitates were also dissolved. Moreover, dispersoids also remained in the microstructure. The SZ area experienced severe plastic deformation and the highest temperature. Therefore, the original primary precipitates could dissolve into the solute and reprecipitate from the supersaturated solid solution during cooling. On the other hand, the HAZ experienced lower temperatures, and there was a lack of shearing. Therefore, the precipitates were only likely to grow due to the additional heat input during FSW. When the BM was already peak hardened to the T6 state, the precipitates in HAZ were likely to increase, leading to a decrease in the microhardness. In [43], the HAZ of AA7075 exhibited significant growth of ƞ′ precipitates, which led to the overaging and softening of this zone compared to BM. Different microhardness levels in the SZ for the weld from CG Al-Mg-Si came from other precipitate types, sizes, and distributions.

The differences in microhardness distributions obtained in the present study are in agreement with microstructure observations and characteristics of second-phase precipitates. For the UFG weld, a drop in the microhardness was caused by grain coarsening and the dissolution of the β″ precipitates in the whole SZ. As both grain size and dissolution occurred in the whole area, this zone was characterized by a homogeneous microhardness distribution. In the case of the CG weld, a drop in the microhardness in the SZ was caused by both—the dissolution of the precipitates and the growth of the β″ to β′/β precipitates, depending on the part of the SZ. As a result, the microhardness distribution in the SZ was not homogeneous. The areas with a lower microhardness correlated with zones with dissolved precipitates, while the areas of the SZ where higher microhardness was observed were those with coarsened precipitates. However, independent of the region, the microhardness was lower compared to BM, where β″ precipitates were present (Figure 5a). For age-hardenable Al alloys, after FSW, a W-shape of microhardness profiles was observed [44], which was connected with the characteristics of precipitates. A similar curve was observed for the CG weld. However, in the case of the UFG weld, the characteristic was different. Based on the results obtained for welds from Al-Mg-Si, postprocessed aging can improve the hardness in the SZ [45], which is connected with the reprecipitation process. For the CG sample, it was beneficial; however, for the UFG BM, it resulted in a recovery of the UFG microstructure and lowered the mechanical strength of this material.

**Figure 8:**
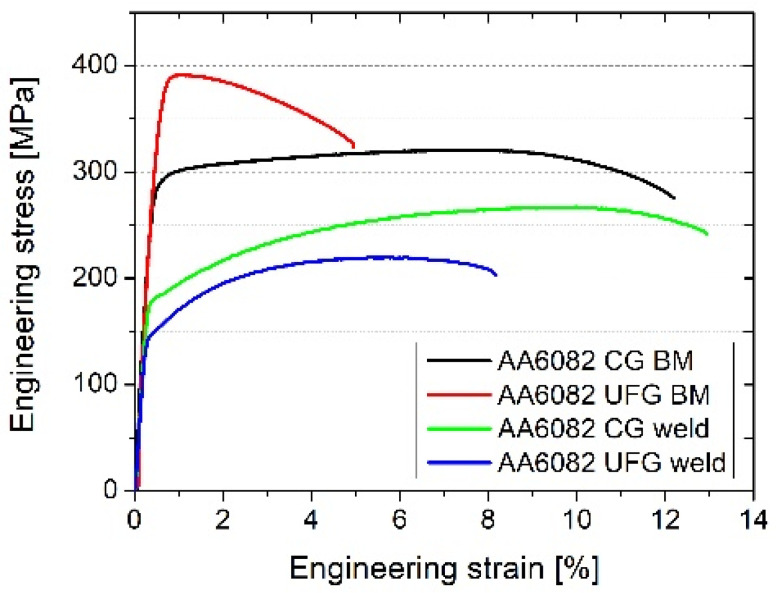
Representative stress–strain curves for the welds and base materials.

**Figure 9:**
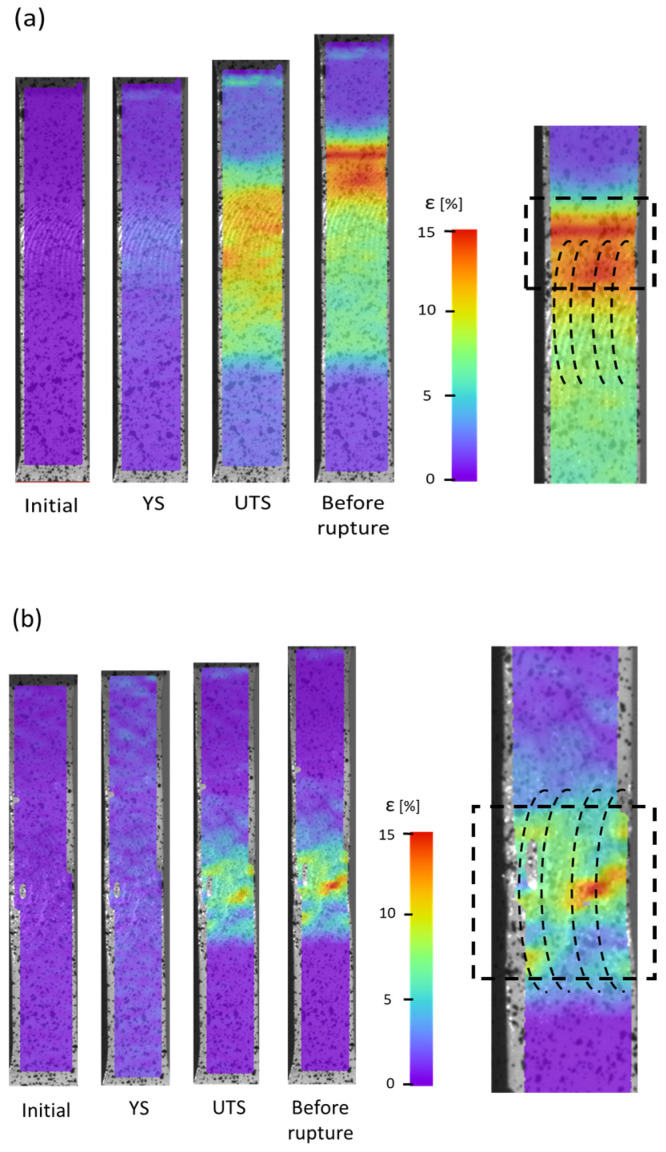
DIC maps of the welds from (**a**) CG and (**b**) UFG Al-Mg-Si alloy.

The results of the tensile tests are presented in Figure 8, where the representative stress–strain curves are shown, and in Figure 9, where DIC straining maps are illustrated. In the case of stress–stress curves, additionally, the results for the BM are presented. The gathered results are shown in Table 3. The initial CG BM sample had a YS of 291 MPa, a UTS of 320 MPa, and E_b_ equaled 13%. After I-ECAP, the strength of the material increased by 70–90 MPa, while the E_b_ decreased to 6%. Such differences were caused by grain refinement and an increase in the number of structural defects, such as dislocations and grain boundaries. According to the modified Hall–Petch equation [46], a decreased average grain size and an elevated number of HAGBs led to an improvement in the strength of the material. For both welds, a decrease in strength compared to BM was observed. The UFG weld had the lowest YS and UTS values. The figures presenting the DIC maps (Figure 9b) show the differences in strain localization between the welds. In the case of the UFG weld, the rupture occurred in the center zone of the SZ, while in the second weld, the break occurred in the area between SZ and TMAZ.

The obtained results of mechanical strength are in agreement with the results of the microstructure. Similar to the microhardness measurements, the values of UTS and YS of the welds were lower compared to the results obtained for the base materials. In the case of the CG weld, the reason for this was the change in the strengthening precipitates—their partial dissolution and partial coarsening. β″ precipitates together with GP zones strengthened the Al-Mg-Si alloy the most significantly [47], and both coarsening and, especially, the dissolution of precipitates resulted in lowering the mechanical strength. For the UFG weld, the lower strength was caused by the dissolution of the precipitates but also grain coarsening, as an increase in grain size caused a decrease in mechanical strength [46]. Another factor could influence the strength of the welds, such as residual stress [48,49]. However, this factor was not investigated in the present study. The rupture location could also be explained via the microstructure, as the rupture occurred in zones with the lowest strength—in the CG weld, it was in the transition between TMAZ and HAZ (which is the region with the lowest microhardness, see Figure 7a), while in the UFG weld, the rupture occurred in the center of the UFG weld. The results of the rupture location proved that the welds were of good quality and that there was a lack of defects because the breakage occurred in the weakest points of the welds.

The obtained results have great potential, as Al-Mg-Si alloy, due to its lightweight, good corrosion resistance, machinability, and weldability, can be used in multiple applications, such as high-stress applications, aircraft and cars components, pipelines, and storage tanks, including hydrogen storage. UFG Al-Mg-Si alloy has enhanced mechanical properties, which may have a positive influence in many applications and further broaden its possibilities. The results obtained from the present study show the direction in which the topic of welding UFG material should be led. The first idea is to implement heat treatment, i.e., aging, after the welding process. However, since the UFG is thermally unstable and prone to recovery and recrystallization, the heat treatment procedure has to be examined carefully. The second area of possible improvement is the FSW process, where additional cooling may lead to less significant grain coarsening in the SZ compared to the UFG BM. This would lead to welds with higher mechanical strength.

## 4. Conclusions

In the present study, the FSW technique was used to join CG and UFG Al-Mg-Si alloy. The welds’ microstructure evolution and its influence on mechanical properties were examined. The following conclusions can be drawn:

- After one pass of I-ECAP, the grain size reduction was obtained from 23 µm to 1.5 µm, while the fraction of HAGB decreased from 95% to 30%. This resulted in a considerable increase in the mechanical strength and microhardness;
- FSW led to changes in the microstructure. In the SZ of both welds, due to dynamic recrystallization, a similar microstructure in terms of grains was obtained with an average size of ~5 µm and a fraction of HAGB of ~75%;
- For both welds in the SZ, a decrease in microhardness was observed compared to the BM. However, a homogeneous drop was observed for the weld from UFG Al-Mg-Si with an average value of 65 HV0.1, while for the weld of CG Al-Mg-Si, an average value of 76 HV0.1 was obtained. Moreover, in the latter weld, a heterogeneous microhardness distribution was obtained;
- The results of the mechanical strength of welds revealed a drop compared to the BM. The rupture occurred in the SZ for the UFG weld and in the TMAZ/SZ for the CG weld.

## Figures and Tables

**Table 1:** Chemical composition of the examined EN AW-6082 alloy [17].

Element	Al	Mg	Si	Cu	Fe	Zn	Mn	Cr
Content [wt.%]	balance	0.60–1.20	0.70–1.30	Max. 0.10	Max. 0.50	Max. 0.20	0.40–1.00	Max. 0.25

**Table 2:** Average grain size values and a fraction of HAGB for SZ and BM of the welds.

Weld Zone	d [µm]	HAGB [%]
CG BM	23.1 ± 14.8	95.1
CG SZ	4.7 ± 2.1	76.7
UFG BM	1.5 ± 1.0	30.5
UFG SZ	5.2 ± 2.6	74.6

**Table 3:** The summary of the results from the tensile test for the welds and BM.

Sample	YS [MPa]	UTS [MPa]	E_b_ [%]
AA6082 CG BM	291 ± 1	320 ± 1	13 ± 1
AA6082 UFG BM	387 ± 3	394 ± 5	6 ± 1
AA6082 CG weld	184 ± 3	269 ± 2	12 ± 1
AA6082 UFG weld	153 ± 4	220 ± 2	9 ± 1

## Data Availability

The data used to support the findings of this study are available upon request.
